# Enzymatic digestibility of lignocellulosic wood biomass: Effect of enzyme treatment in supercritical carbon dioxide and biomass pretreatment

**DOI:** 10.1016/j.heliyon.2023.e21811

**Published:** 2023-10-31

**Authors:** Pawan Kumar, Azadeh Kermanshahi-pour, Satinder Kaur Brar, Chunbao Charles Xu, Quan Sophia He, Sara Evans, Jan K. Rainey

**Affiliations:** a Biorefining and Remediation Laboratory, Department of Process Engineering and Applied Science, Dalhousie University, Halifax, Nova Scotia B3 J 1Z1, Canada; b Department of Civil Engineering, Lassonde School of Engineering, York University, North York, Toronto, Ontario M3J 1P3, Canada; c School of Energy and Environment, City University of Hong Kong, Hong Kong SAR, Hong Kong; d Department of Engineering, Faculty of Agriculture, Dalhousie University, Truro, Nova Scotia B2N 5E3, Canada; e Department of Chemistry, Dalhousie University, Halifax, Nova Scotia B3H 4R2, Canada; f Department of Biochemistry & Molecular Biology and School of Biomedical Engineering, Dalhousie University, Halifax, Nova Scotia B3H 4R2, Canada

**Keywords:** Lignocellulosic biomass, Biomass pretreatment, Enzyme pretreatment, Supercritical CO_2_, Cellulase, Enzymatic hydrolysis, Fermentable sugars

## Abstract

Energy and resource intensive mechanical and chemical pretreatment along with the use of hazardous chemicals are major bottlenecks in widespread lignocellulosic biomass utilization. Herein, the study investigated different pretreatment methods on spruce wood namely supercritical CO_2_ (scCO_2_) pretreatment, ultrasound-assisted alkaline pretreatment, and acetosolv pulping-alkaline hydrogen peroxide bleaching, to enhance the enzymatic digestibility of wood using optimized enzyme cocktail. Also, the effect of scCO_2_ pretreatment on enzyme cocktail was investigated after optimizing the concentration and temperature of cellulolytic enzymes. The impact of scCO_2_ and ultrasound-assisted alkaline pretreatments of wood were insignificant for the enzymatic digestibility, and acetosolv pulping-alkaline hydrogen peroxide bleaching was the most effective pretreatment that showed the release of total reducing sugar yield (TRS) of ∼95.0 wt% of total hydrolyzable sugars (THS) in enzymatic hydrolysis. The optimized enzyme cocktail showed higher yield than individual enzymes with degree of synergism 1.34 among the enzymes, and scCO_2_ pretreatment of cocktail for 0.5–1.0 h at 10.0–22.0 MPa and 38.0–54.0 °C had insignificant effect on the enzyme's primary and global secondary structure of cocktail and its activity.

## Highlights

• Wood and enzyme pretreatment performed separately prior to enzymatic hydrolysis.
• ScCO_2_ and alkali assisted-ultrasonic methods were ineffective for wood pretreatment.
• Short exposure (0.5–1.0 h) of enzyme to scCO_2_ does not affect the enzyme activity.
• Sugar yield of ∼95.0 wt% was achieved in enzymatic hydrolysis by acetosolv pulping.

## Graphical abstract

**Figure:**
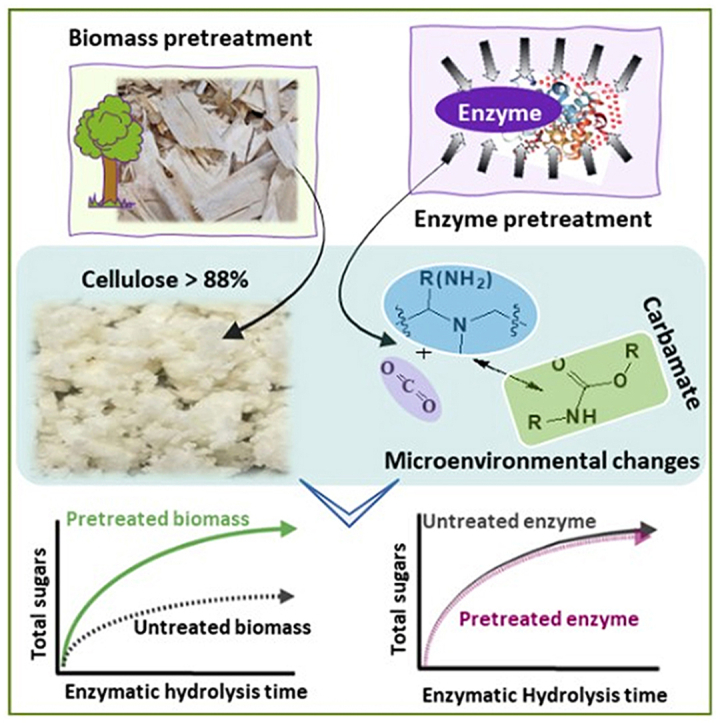

## 1 Introduction

Lignocellulosic (LC) biomass is one of the most abundant sources for the production of commodity and value-added chemicals. However, the polymeric network of cellulose, hemicellulose and lignin is complex and resistant to conversion to monomeric components [1,2]. Biomass must go through different pretreatment processes to disrupt the recalcitrant structure to enhance the saccharification of polysaccharides (cellulose & hemicellulose) to reducing sugars (cellobiose, glucose from cellulose, xylose, mannose, arabinose from hemicellulose) by enzymatic hydrolysis. Agricultural biomass has been studied more extensively compared to woody biomass. Conventional pretreatment including Kraft or sulfite pulping and chlorinated bleaching methods are effective in enhancing the enzymatic digestibility of biomass [[3], [4], [5], [6]]. However, there is significant environmental impact associated with these conventional methods due to the use of toxic chlorinated compounds, also known as adsorbable organic halides [7,8]. A performance evaluation based study showed that the combination of methods such as alkali-peroxide, mechanical and enzymatic treatment, is less energy intensive and generate lower amount of waste compared to conventional biomass pretreatment methods such as chemical soaking and thermochemical treatments [[9], [10], [11]]. Although the use of chemicals is not eliminated in these methods, the amount of hazardous chemicals and therefore the environmental impact associated with hazardous waste management are reduced. Therefore, it is very worthwhile to explore such non-conventional method on wood biomass. Among the variety of such pretreatment methods available, ultrasound, organosolv, and supercritical CO_2_ (scCO_2_) pretreatment are emerging as physicochemical pretreatment methods with the advantage of reduced wastewater generation and potential for solvent recyclability [[12], [13], [14], [15]]. Organosolv pulping using organic solvents and acids such as ethanol, formic acid, and acetic acid in the presence of mineral acids (i.e., sulfuric acid, hydrochloric acid) to catalyze the breaking of the ether bonds of lignin and initiate solubilization. The process loosens the biomass structure exposing the polysaccharides [[16], [17], [18]]. Ultrasound pretreatment in an alkaline aqueous medium produces hydroxyl radicals and results in high temperature and extreme shear force due to cavitation, which breaks linkages in the lignin and hemicellulose network [19,20]. In addition, an alkaline medium leads to a lower extent of sugar degradation compared with acid or thermal pretreatments [21]. The scCO_2_ as a pretreatment solvent, has the advantage of high diffusivity and low viscosity, penetrating the moistened LC biomass solids and forming unstable carbonic acid, which catalyzes hemicellulose hydrolysis. Additionally, scCO_2_ disrupts the polymeric network of biomass by an explosion effect upon rapid pressure release [22,23].

The polysaccharides in the pretreated biomass are hydrolyzed to monomeric and oligomeric sugars by cellulolytic enzymes. Cellulase enzyme is a mixture of endoglucanase, exoglucanase, and β-glucanase enzymes, which are produced by a variety of microbial species. Reducing the enzyme loading and enhancing the enzymatic hydrolysis rate are the strategies to improve the process economics. The synergistic effect of enzymes increases the rate of hydrolysis by changing hydrolysis patterns [24]. For example, cellulose hydrolysis mechanisms of two enzymes (free cellulase cocktail named Ctec2 and cellulosomes or self-assemblies of macromolecular enzyme complex) were observed by transmission electron microscope. The free cellulase hydrolyzed from the edges of cellulose fibers, whereas the cellulosome separated the cellulose microfibrils and increased the surface area. The avicel cellulose was completely hydrolyzed in 24 h using cocktail of enzymes whereas the free cellulase and cellulosome enzymes hydrolyzed ∼70 % and ∼100 %, respectively, of cellulose in 48 h when used separately [25]. Another emerging strategy for enhancing enzymatic hydrolysis rate or/and yield is the pretreatment of an enzyme under scCO_2_. It has been reported in recent studies that enzymes exposed to scCO_2_ have shown improved stability and activity in free and immobilized conditions [[26], [27], [28], [29], [30], [31]].

This study explores a greener and more sustainable process with mild reaction conditions for processing LC biomass resources, which could significantly reduce the discharge of contaminated wastewater from lignocellulosic biorefineries. The goal of this study is to explore the use of scCO_2_ as a greener and less chemical-based pretreatment method to understand its impact on the enzymatic digestibility of spruce wood. In the course of this study, we have specifically developed an understanding on the effect of the scCO_2_ treatment of cellulose-degrading enzyme cocktails on enzyme activity and performance in hydrolysis of spruce wood. Therefore, the current work specifically investigated the effect of scCO_2_ pretreatment, ultrasound-assisted alkaline pretreatment, and acetosolv pulping-alkaline peroxide pretreatment of spruce wood biomass on its enzymatic digestibility for total reducing sugar (TRS) production. Improved enzymatic activity has been reported for scCO_2_-treated enzymes and is considered as an emerging method for enhancing enzyme activity [32]. Therefore, the effect of scCO_2_ pretreatment of enzyme cocktail on enzymatic hydrolysis of pretreated wood for the total reducing sugar (TRS) production was also investigated. Exploration of such pretreatment methods could substantially reduce the use of chemicals and wastewater generation that could help to approach a more sustainable process [33,34].

## 2 Materials and methods

### 2.1 Materials

Spruce wood chips were ground using a coffee grinder (Hamilton Beach, Canada) and screened to a particle size range of 0.5–1.0 mm with ASTM mesh screens #18.0/35.0. Extractives from the biomass were removed by a Soxhlet extraction apparatus using distilled water followed by a toluene-ethanol mixture (2.0:1.0, v/v) separately for 6 h each. The extractives-free biomasses were dried at 105.0 °C in an oven under an active vacuum. Liquid CO_2_ (99.9 % with eductor) was sourced from Praxair, Inc.

Cellulase (lyophilized powder of purified enzyme) from *Trichoderma reesei* ATCC 26921 (C8546–10KU) and cellulolytic enzyme complex Viscozyme L (solution containing xylanases, pectinases, β-glucanase enzymes along with high concentration glucose and xylose sugars) from *Aspergillus aculeatus* (V2010-50 ML), and all chemicals were obtained from Sigma-Aldrich unless otherwise noted. Cellulase powder was dissolved in distilled water at 15.0 g/L. Sodium citrate buffer (1.0 M) was prepared by mixing citric acid monohydrate and sodium hydroxide, diluted to 10.0 mM, and pH was adjusted to 5.0 using sodium hydroxide [35]. 0.002 % (w/v) sodium azide was used as an antimicrobial agent [36]. Enzymes were used without further purification and enzymatic reactions were conducted at pH 5.0 of 10 mM concentration citrate buffer to facilitate hydrolytic activity of enzymes. Cellulase and Viscozyme L were mixed at an optimum concentration determined by the response surface method (RSM) to prepare the enzyme cocktail to be used in the enzyme hydrolysis reaction (detailed in supplementary information section 2).

### 2.2 Analytical methods

The protein content of enzyme solutions was determined by colorimetric assay using Pierce™ bicinchoninic acid (BCA) reagents, (Thermo Scientific) using bovine serum albumin (BSA) for calibration curve development [37]. The reducing sugars were analyzed by high-performance liquid chromatography (HPLC) with a refractive index detector (RID) (Agilent 1260 infinity II) with water as mobile phase at 0.6 mL/min, Agilent Hi-plex H (300.0 × 7.7 mm, particles size 8.0 μm) at temperature 65.0 °C, and RID temperature at 55.0 °C.

### 2.3 Chemical composition of biomass

The total hydrolyzable sugars (THS) in the wood samples were determined by their compositions using national renewable energy laboratory (NREL) standard method for carbohydrate and lignin analysis before and after the pretreatments [38]. The biomass was dried in the oven at 105.0 °C for 6.0 h under an active vacuum to determine the moisture content. Further, 0.3 g of dry solid were treated with 3.0 mL of 72 wt% sulfuric acids in a pressure tube for 1.0 h at 30.0 °C in a water bath. After 1.0 h, 83.0 mL water was added to the reaction tube to reach an acid concentration of 4.0 wt%. The reaction mixture was autoclaved (Sterilmatic Market Forge) for 1.0 h at 121.0 °C and 0.1 MPa (15.0 psi). The solution was cooled to ambient temperature and filtered using an 11.0 μm glass filter to separate the insoluble lignin. The insoluble lignin on the glass filter was dried by oven at 105.0 °C for 6.0 h under an active vacuum. The filtrate was neutralized to pH 5.0–6.0 using calcium hydroxide, filtered using a 0.2 μm syringe filter and analyzed by HPLC-RID [16].

### 2.4 Pretreatment of lignocellulosic biomass

#### 2.4.1 Supercritical CO_2_ pretreatment

**Fig. 1:**
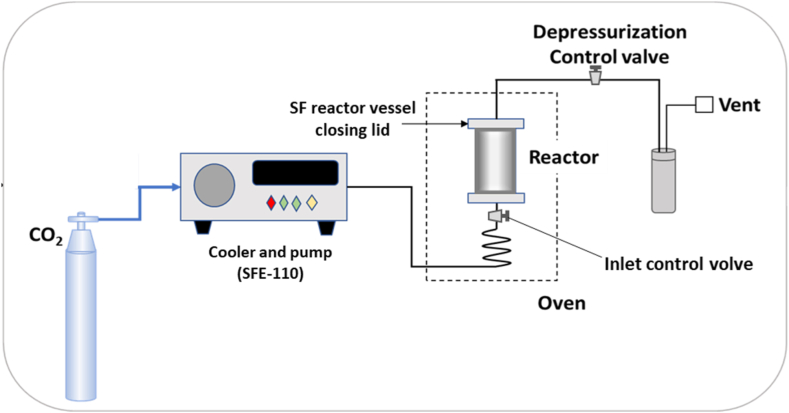
Schematic diagram of supercritical CO_2_ reactor configuration.

Spruce wood was pretreated with scCO_2_ at different temperatures (100.0, 140.0, and 180.0 °C) and times (0.5, 1.0, 2.0, and 4.0 h) under pressure (15.0, 20.0, and 25.0 MPa) with a water to solid ratio of 2.0 mL/g (2.0 mL water with 1.0 g wood) in a 20.0 mL scintillation vial. Further, the effect of water solid ratio was investigated in the range of 1.0,2.0, 4.0, and 10.0 mL water per g wood. The scintillation vial containing water and biomass was placed in the 100 mL supercritical fluid reaction vessel and closed the reactor (Fig. 1). The reactor vessel was pressurized with liquid CO_2_ after preheating the reactor. The CO_2_ inlet and outlet valve were closed, and the reaction was carried out in static conditions. After supercritical CO_2_ pretreatment, the reactor was depressurized rapidly (3.0–4.0 MPa/min). The pretreated wood was dried and resuspended in 10 mL distilled water to determine the hydrolyzed sugars during the scCO_2_ pretreatment. Before the enzymatic digestion, the pretreated wood was washed with distilled water followed by drying in an oven at 105.0 °C for 6.0 h under an active vacuum.

#### 2.4.2 Ultrasound pretreatment

Spruce wood samples (2.0 g) were pretreated with an ultrasound probe (Sonics ultrasonic processor, VCX750, 13.0 mm probe diameter) at 90.0 % amplitude for 20.0 min in 40.0 mL of 2.0 % w/v NaOH solution without temperature control. To evaluate the effect of NaOH, 2 g wood was treated in 40.0 mL of 2.0 % w/v NaOH solution at 70.0 °C for 20.0 min at 125.0 rpm (revolutions per minute) without any ultrasound treatment as control. About 2.0 g wood was also treated with an ultrasonic probe at 90.0 % amplitude for 20.0 min in 40.0 mL water instead of 2.0 % w/v NaOH solution as the second control. After pretreatment, wood samples were adjusted to a neutral pH (6.5–7.0) by washing with distilled water.

#### 2.4.3 Acetosolv pulping followed by alkaline-peroxide bleaching pretreatment

The extractive-free spruce 20.0 g was pretreated using 198.6 mL of 92.5 % w/w acetic acid in the presence of 1.4 mL hydrochloric acid catalyst (0.3 % w/w) for 3.0 h at 115.0 ± 2.0 °C. The pulp obtained was bleached by 13.5 % w/w hydrogen peroxide in 4.0 % w/v sodium hydroxide solution for 2.5 h at 50.0 °C [16]. The pulp from acetosolv pulping and bleaching was then adjusted to neutral pH (pH 6.5–7.0) by washing with distilled water and dried in the oven at 55.0 °C under active vacuum for 24.0 h as described in Ref. [16].

### 2.5 Supercritical CO_2_ pretreatment of enzyme

The enzyme cocktail (cellulase 1.94 mg with cellulolytic complex enzymes 131.0 mg) in sodium citrate buffer was pretreated with supercritical CO_2_ (8.0 mL enzyme cocktail in a 20.0 mL scintillation vial) in a 100.0 mL reactor vessel (Fig. 1). The reactor was pressurized to set pressure with liquid CO_2_ at set temperature, the effects of temperature (38.0–54.0 °C), pressure (10.0–22.0 MPa), and holding time (30.0–60.0 min) were examined. The reactor was depressurized at a slow rate of 0.3–0.4 MPa/min after the holding time. The enzyme cocktail was also pretreated for 24.0 and 48.0 h time at 46.0 °C, 16.0 MPa to investigate the effect of exposure of enzyme cocktail to scCO_2_ for a longer time.

### 2.6 Enzymatic hydrolysis

Each of the untreated and pretreated wood were subjected to enzymatic hydrolysis in 25.0 mL Erlenmeyer flasks at 150.0 rpm in a Corning LSE Benchtop Shaking Incubator. Enzymatic hydrolysis reactions were performed at 2 % (w/v) substrate in 10 mL sodium citrate buffer (10.0 mM, pH 5.0) containing 0.002 % (w/v) sodium azide as an anti-microbial agent. The enzyme loads per g biomass for each enzyme separately as well as for the enzyme cocktail, and temperature were optimized at a range of concentrations (5.9–38.8 mg cellulase/g spruce wood and 44.4–625.4 mg Viscozyme L/g spruce wood) and temperatures (30.0–55.0 °C) by response surface method (RSM) (supplementary information). During enzymatic hydrolysis, samples were withdrawn from the reaction over time to investigate the hydrolysis profile. Specifically, the yield of reducing sugars was determined using Eq. 1.0 where total reducing sugar (TRS) is represented by the sum of the cellobiose, glucose, and xylose released in reaction. The residual activity of the enzyme after the scCO_2_ pretreatment was calculated using Eq. 2.0.

(1) $$TRS\;yield\;\left(wt.\%\;of\;THS\right)=\frac{total\;released\;sugar\;\left(mg\right)}{\frac{cellulose\;in\;feedstock\;\left(mg\right)}{0.9}+\frac{hemicellulose\;\left(mg\right)}{0.88}}\times 100$$

(2) $$Residual\;activity\;\left(\%\right)=\frac{Sugar\;yield\;by\;pretreated\;enzyme}{Sugar\;yield\;by\;untreated\;enzyme}\times 100$$

For comparison of the enzyme load, the enzyme activity was determined by filter paper hydrolysis at optimum temperature of enzyme. One filter paper activity unit (FPU) is defined by release of 1.0 μmol of reducing sugars per min per mg of enzyme in 1 h reaction. The synergy between the two enzymes was evaluated following by Andersen et al. (2008) using Eq. 3.0 where the enzymatic reactions with cellulase, cellulolytic enzyme complex, and the cocktail were conducted under their optimum conditions [39,40].

(3) $$Degree\;of\;synergism=\frac{{Extent\;of\;conversion}_{Cellulase+cellulolytic\;enzyme\;complex}}{{Extent\;of\;conversion}_{Cellulase}+{Extent\;of\;conversion}_{cellulolytic\;enzyme\;complex}}$$

### 2.7 Structural characterization of enzyme

#### 2.7.1 Primary structure analysis

The effect of scCO_2_ on the primary structure enzymes was analyzed using SDS-PAGE (sodium dodecyl sulfate–polyacrylamide gel electrophoresis). The treated and untreated enzyme (cellulase, cellulolytic complex enzymes, and cocktail) protein samples were prepared by mixing equal volumes of 4.0 mg/mL protein sample and 2.5 × SDS-PAGE reducing loading buffer and heated at 90.0 °C for 10.0 min. The samples and a molecular weight ladder (Precision Plus Protein Unstained Stained Standards, Bio-Rad Laboratories) were resolved by electrophoresis using a 15.0 % sodium dodecyl-sulfate polyacrylamide gel (200.0 V for 50.0 min) and visualized by staining with Coomassie Brilliant Blue R-250.

#### 2.7.2 Secondary structure analysis

Fourier transform infrared (FTIR) spectra were collected (32.0 scans, 4.0 cm^−1^ resolution, range of 4000.0 to 700.0 cm^−1^) using a Nicolet iZ10 spectrometer (Thermo Fisher Scientific) equipped with a liquid nitrogen cooled mercury cadmium telluride (MCT) detector (Thermo Fisher Scientific) and a ConcentratorIR2 Multiple Refraction Attenuated Total Reflection (ATR) attachment with a Silicon ATR crystal (Harrick Scientific Products Inc.) at room temperature (22.5 ± 2.5 °C). 15.0 μL of each protein sample in 20.0 mM sodium citrate buffer at pH 5.0 were deposited on the ATR crystal immediately prior to data acquisition. Data collection and analysis were performed using Omnic version 9.11.745 (Thermo Fisher Scientific) with OriginPro version 10.0.0.154 used for visualization. Following baseline correction, the amide I region (1600.0–1700.0 cm^−1^) was deconvoluted to evaluate protein secondary structure using Byler and Susi (1986) for wavelength attributions to secondary structure [41].

Circular dichroism (CD) spectra were acquired using an Olis DSM20 CD Spectrophotometer with integration time determined as a function of High Volts in Olis SpectralWorks Version 5.888.272. CD spectra were acquired at room temperature (22.5 ± 2.5 °C) from 270.0 to 180.0 nm with a 1.0 nm step size using quartz cuvettes of 0.01 mm path length (Hellma). Spectra were obtained by averaging three individual scans. The spectra of 20.0 mM sodium citrate blanks were measured before the samples and were subtracted from the 0.1 mg/mL protein sample CD spectra. Since the samples are mixtures of proteins and, hence, molarity is not known, to directly compare from sample to sample each spectrum was normalized to its respective minimum in the far-UV regime [42]. The secondary structure was analyzed using two popular method FTIR and CD spectra to provide a qualitative difference between the results.

### 2.8 Statistical analysis

All the experiments were performed using at least independent duplicates. The Data was analyzed with one way-ANOVA at 95 % confidence level (p < 0.05) for the statistical significance. A paired *t*-test (P < 0.05) was used to analyze the significances of different pretreatment conditions of enzyme pretreatment on TRS yield. The statistical analyses were performed in Minitab® 21.4.

## 3 Results and discussion

### 3.1 Chemical composition of spruce wood and enzymes

The chemical composition of the untreated and pretreated spruce wood was analyzed using the NREL method and the compositions are shown in Table 1. The spruce wood polysaccharide content is 65.0–70.0 wt% similar to reports in the literature [43,44]. The scCO_2_ treatment of the untreated wood did not have a significant influence on the chemical composition of the solids. The ultrasound-assisted alkaline pretreatment of the wood showed a reduction in total polysaccharide component from 67.0 wt% to 54.0 wt% whereas lignin content slightly increased from 29.0 to 32.0 wt% because of polysaccharide decomposition (Table 1). The polysaccharide content was increased from 65.0-67.0 % to 95.0–96.0 % after acetosolv pulping followed by alkaline hydrogen peroxide bleaching treated wood, a process which removed most of the lignin. The wood pulp derived from acetosolv pulping followed by alkaline-peroxide bleaching was also pretreated with scCO_2_ and no significant change in the chemical composition was observed (data not shown).

**Table 1:** Chemical composition of untreated and pretreated spruce wood in wt.% (n = 3).

Biomass Component	Untreated biomass	scCO_2_ pretreatment (20.0 MPa, 180.0 °C, 1.0 h, 2.0 g/mL)	Ultrasound-assisted 2.0 wt% NaOH pretreatment	Pulping-bleaching pretreatment
Cellulose	47.1 ± 2.2	39.7 ± 0.8	36.2 ± 0.7	88.5 ± 0.7
Hemicellulose	20.1 ± 0.8	19.3 ± 0.9	17.8 ± 0.7	6.2 ± 0.7
Lignin	29.4 ± 0.2	31.1 ± 0.6	29.4 ± 2.9	<0.7

Before enzymatic hydrolysis of the untreated and pretreated biomass, the commercial enzymes cellulase and Viscozyme L were used to prepare the enzyme cocktail and characterized for their sugar and protein content. Sugar and protein were analyzed by HPLC and BCA protein assay, respectively (Table 2). Since the cocktail is made up of a crude enzyme complex solution (Viscozyme L), the protein concentrations of cocktail are substantially high in comparison to cellulase from *T. reesei*. The prepared enzyme cocktail using the commercial enzymes was used without further filtration, and therefore, an initial sugar concentration of 15.2 ± 0.4 g/L was present in the reaction mixture at the beginning of the reaction. However, there was no significant influence of initial sugars concentration on the activity and TRS yield (Fig. S6).

**Table 2:** The commercial enzyme (as received) characterization for sugar and protein content (n = 3).

Commercial enzyme	Sugar concentration	Protein concentration	Enzyme activity (FPU/g), temperature (°C), pH
Cellulase	None	1.2 ± 0.0 g dissolved protein/g enzyme powder	75.2 ± 0.4, 46.3, 5.0
Cellulolytic complex enzymes (Viscozyme L)	289.7 ± 6.8 g/L	210.8 ± 8.4 g/L enzyme solution	23.0 ± 3.3, 37.4, 5.0

### 3.2 Optimum concentration and temperature of individual enzymes in the cocktail mixture

Optimal enzyme concentration (cellulase 5.9–34.1 mg, cellulolytic complex enzymes 44.4–256.5 mg) and temperature (30.0–58.0 °C) were investigated using TRS yield (wt.% of total hydrolyzable sugars) as a response in RSM (Tables S1–S8). The TRS yields from wood by individual enzymes showed that cellulase has an optimum temperature of 46.3 °C and cellulolytic complex enzymes have 37.4 °C (Figs. S1 and S2) in sodium citrate buffer (10.0 mM, pH 5.0). The differences in the optimum temperature and activities of enzymes are highly dependent on the source microorganism growth and reaction conditions [[45], [46], [47]]. The optimum concentrations of these commercial enzymes in the enzyme cocktail were 9.7 mg cellulase and 598.4 mg cellulolytic complex enzymes per gram of untreated spruce wood substrate at 2.0 wt% substrate concentration in 10.0 mL sodium citrate buffer (10.0 mM, pH 5.0) at 42.5 °C and 150.0 rpm. Under optimized condition for the enzyme cocktail, the untreated wood showed a maximum total reducing sugars (TRS) yield of 14.1 ± 0.8 wt% of total hydrolyzable sugar (THS) in 72.0 h at atmospheric pressure (Figs. S3 and S4).

**Fig. 2:**
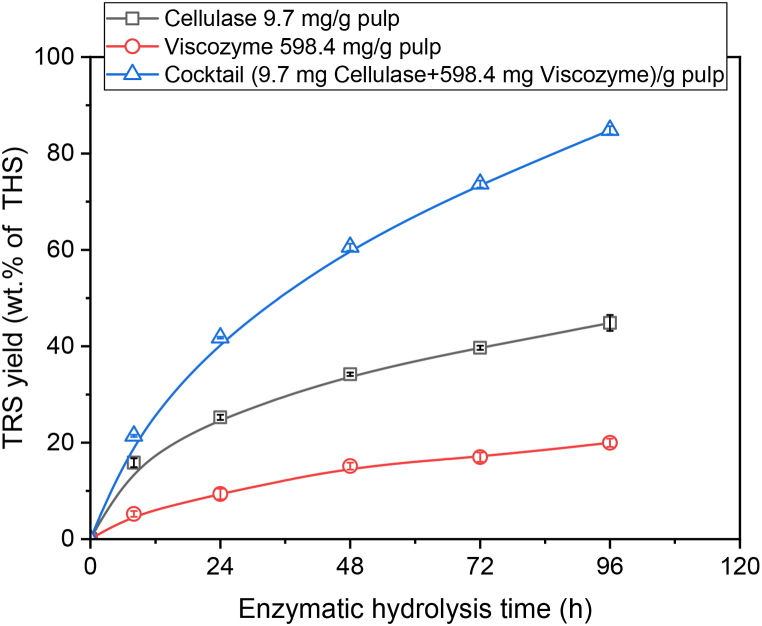
Synergistic effect between the cellulase and cellulolytic complex enzymes for TRS yield from bleached wood pulp over time (Cellulase 9.7 mg/g pulp, Viscozyme 598.4/g pulp separately as well as combined in cocktail) (n = 2).

Under the same optimized reaction conditions of cocktail, the bleached wood pulp (BWP) showed a TRS yield of 74.9 ± 0.8 wt% of THS in 72.0 h. Therefore, BWP was used as a substrate for the enzyme cocktail to investigate the synergy between the two enzymes present in the cocktail. The BWP was hydrolyzed by individual enzymes at the same amount as is present in the enzyme cocktail and the reaction was carried out at their optimum temperatures for 96.0 h. In individual runs, cellulase released 44.9 wt% TRS at 46.3 °C and cellulolytic complex enzymes released 20.0 wt% TRS at 37.4 °C whereas the TRS yield increased to 86.9 ± 1.78 wt% by the enzyme cocktail in 96.0 h at 42.5 °C. The synergism between enzymes was 1.3 showing the performance of the enzyme cocktail to be 34.0 % higher than individual component's performance (Fig. 2). The Viscozyme L commercial enzyme was used as hemicellulase equivalent enzymes hydrolyzes arabinan and xylan from the hemicellulosic network [48,49]. Endoglucanase has also been found in Viscozyme L cellulolytic complex enzymes which hydrolyzes the glucan chains in cellulose from the interior and releases short chains of glucan [50]. The exposed chains of cellulose are easily accessible for exoglucanase and β-glucanase enzymes that release cellobiose and glucose monomers. Simultaneously, hemicellulase also acts on hemicellulose chains and releases hemicellulosic monomers (i.e., xylose, mannose). The presence of these enzymes in the enzyme cocktail released around ten times more xylose than the cellulase alone and synergism between enzymes led to higher TRS yield.

### 3.3 Effects of pretreatments on spruce wood

The pretreated wood was enzymatically digested with the enzyme cocktail under its optimized reaction condition (2.0 % solid, enzyme cocktail: Cellulase 9.7 mg/g pulp, Viscozyme 598.4/g pulp biomass, 42.5 °C) and compared with the TRS yield from untreated biomass.

#### 3.3.1 Effect of supercritical CO_2_ pretreatment on spruce wood

**Fig. 3:**
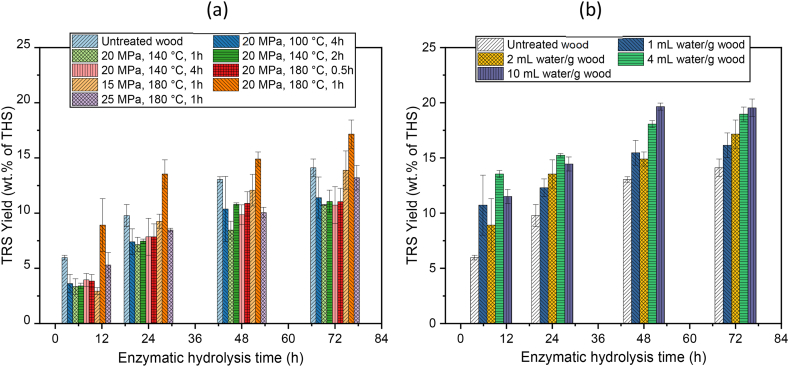
(a) Effect of scCO_2_ pretreatment (Pressure, Temperature, holding time) keeping the water-solid ratio constant at 2.0 mL/g and (b) effect of water solid ratio at 180.0 °C, 20.0 MPa for 1.0 h scCO_2_ pretreatment on spruce wood for TRS yield in enzymatic hydrolysis (n = 2).

The effectiveness of the scCO_2_ pretreatment on spruce wood was analyzed by enzymatic digestibility of pretreated solids using an enzyme cocktail under optimized conditions. The spruce wood was pretreated with 2.0 mL water per g wood (dry weight) under 20.0 MPa pressure at temperatures 100.0–180.0 °C for 0.5–4.0 h holding time. The total reducing sugar of 4.0 wt% of THS at maximum were released at high temperature (180.0 °C) and 10.0 mL/g water-solid ratio whereas lower temperature (100.0–140.0 °C) did not show any detectable amount of sugar during the scCO_2_ pretreatment. The temperatures below 180.0 °C were not able to disrupt the recalcitrant structure of wood and the TRS yield from pretreated wood was similar to untreated wood (Fig. 3). The increase in the water-solid ratio from 1.0 mL/g to 10.0 mL/g in scCO_2_ pretreatment at the respective temperature and pressure of 180.0 °C and 20.0 MPa, had shown slightly increased yield from 16.1 ± 1.8 wt% to 19.5 ± 1.0 wt% at 72.0 h in the subsequent stage of enzymatic hydrolysis for pretreatment. The effect of pretreatment was also done on agricultural biomass cornstalk to verify the instrumental setup. The enzymatic digestibility of scCO_2_ pretreated cornstalk (20.0 MPa, 170.0 °C, 2.5 h) showed slightly improved TRS yield from 21.89 ± 2.9 wt% (untreated) to 29.3 ± 2.6 wt% of THS at hydrolysis time of 96.0 h at water-solid ratio of 0.5 mL/g whereas increasing the water-solid ratio to 4.0 or 10.0 mL/g did not enhance the enzymatic digestibility of pretreated cornstalk (Fig. S5, supplementary information).

Under the same condition of pretreatment, Yin et al. (2014) observed an increase from 16.6 wt% (untreated cornstalk) to 46.4 wt% of THS for pretreated cornstalk in 72.0 h at water-solid ratio of 0.5 mL/g, however, effect of water to solid ratio was not investigated [22]. A few studies have reported that scCO_2_ pretreatment enhanced enzymatic digestibility, showing 27.3–84.7 wt% of THS yield from different wood biomass (i.e., aspen wood 84.7 % [51], Southern yellow pine 27.3 % [51], mixed hardwood 73.0 % [52]). However, the reported yields vary significantly depending on the biomass type and pretreatment conditions [53]. For example, aspen wood was pretreated with scCO_2_ (21.4 MPa, 165.0 °C, 0.5 h) with a water to solid ratio of 0.7 mL/g leading to 84.7 wt% of THS yield in enzymatic hydrolysis. Whereas only 27.3 wt% of THS yield was obtained in the enzymatic hydrolysis of scCO_2_ pretreated southern yellow pine under the same condition as the aspen wood [51]. The higher yield observed with hardwood biomass could be related to its distinct chemical composition as well as to the pretreatment conditions employed. The high temperature with scCO_2_ conditions hydrolyzes the hemicellulose fraction and reduces the crystalline structure of biomass which improves the enzyme-carbohydrate interaction in the pretreated biomass [51,52]. Another major reason could be the presence of higher lignin content which may form non-productive binding with enzyme and reduce enzyme-carbohydrates interaction [54,55]. The lower lignin content in hardwood compared to softwood may lead to substantial differences in the enzymatic hydrolysis yield after the scCO_2_ pretreatment. The collective effect of lignin content and hemicellulose hydrolysis during pretreatment could be attributed to the substantial difference in the yield softwood compared to yield from hardwood [51,52]. However, woody biomass is generally under-explored as a LC biomass for scCO_2_ pretreatment; therefore, further investigation with respect to a comparative study of softwood and hardwood biomass from different species would provide greater insight into the effect of wood type-dependent chemical composition on hydrolysis yield.

After sub and supercritical CO_2_ pretreatment, agricultural biomass of various species had shown a TRS yield of 42.0–97.8 wt% of THS upon enzymatic hydrolysis (i.e., sugarcane bagasse 97.8 % [56], switchgrass 42.0–80.0 % [23,52,57], corn stover 66.5–85.0 % [23,52], Guayule 82.8–86.0 % [58,59]). For example, Phan & Tan (2014) used scCO_2_ pretreatment of sugarcane bagasse (15.6 MPa, 187.0 °C, 0.67 h) followed by hydrogen peroxide treatment before enzymatic hydrolysis of the pretreated solid that yielded 97.8 wt% of THS whereas untreated biomass had a yield of only 13.4 wt% of THS [56]. Luterbacher et al. (2010) obtained a yield that increased from 53.0 ± 2.0 wt% (untreated corn stover) to 85.0 wt% of THS yield in enzymatic hydrolysis of scCO_2_ pretreated (20.0 MPa, 160.0 °C, 1.0 h) corn stover biomass without any additional pretreatment [52].

#### 3.3.2 Effect of ultrasound-assisted alkaline pretreatment

**Fig. 4:**
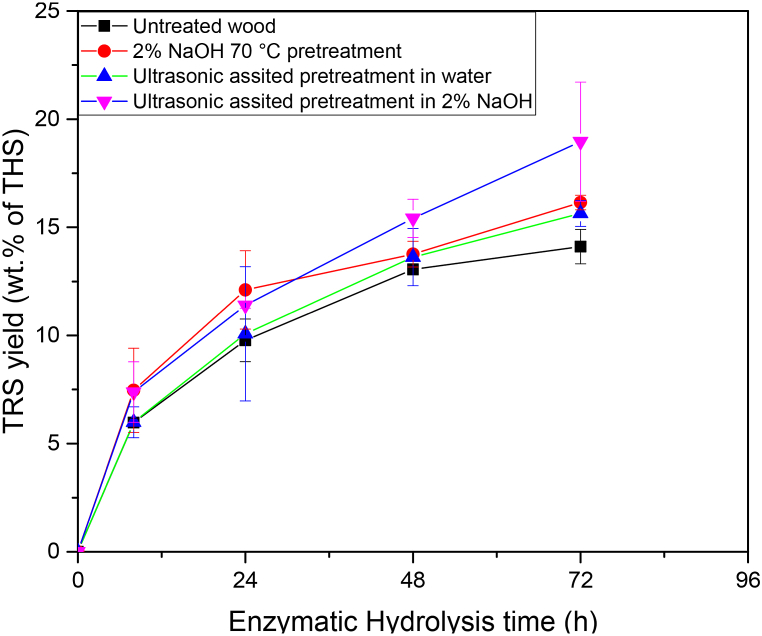
Effect of alkali and ultrasound-assisted pretreatments of wood for TRS yield in enzymatic hydrolysis (n = 2).

Alkaline pretreatment at low alkali concentration (2.0 wt% NaOH) at 70.0 °C for 20.0 min, ultrasound-assisted alkaline pretreatment, and ultrasound pretreatment without an alkaline solution were performed to investigate the effect of ultrasound treatment and alkaline pretreatment. Ultrasound pretreatment of wood in an aqueous solution of 2.0 % NaOH showed an increased yield (20.0 ± 2.8 wt% of THS) in enzymatic hydrolysis in comparison to the yield from untreated wood (14.1 ± 0.8 wt% of THS) in 72.0 h. Pretreatment with alkaline conditions and ultrasound pretreatment without alkaline conditions both led to slightly increased TRS yield in enzymatic hydrolysis in comparison to untreated wood (Fig. 4). However, the increase in the TRS yields was statistically significant (p = 0.13). The ultrasound treatment generates hydroxyl radicals from water, high temperature, and extreme shear force due to cavitation which helps in the deconstruction of the lignin and hemicellulose network [19,20]. However, a lower concentration of sodium hydroxide at low temperatures (rose to 70.0 °C by the 20.0 min ultrasound treatment, measured by thermocouple) and lower treatment duration (20.0 min) was not sufficient to facilitate enhanced enzymatic digestibility, with the spruce wood polymeric structure remaining intact. The chemical composition of the spruce before and after the pretreatment was compared (Table 1) and it was found that the lignin content remained the same whereas the cellulose and hemicellulose contents were hydrolyzed partially during ultrasound-assisted alkaline pretreatment of wood. Similar compositional results (hemicellulase decreased from 20.8 to 13.9 %, and lignin remained at 28.3 to 27.6 %) were obtained for spruce wood after pretreatment with 7 wt% sodium hydroxide at −15.0 to 100.0 °C for 2.0 h but cellulose content increased (from 43.0 to 50.6 %) in all the conditions without ultrasound treatment [60]. Increasing the concentration of sodium hydroxide and the treatment duration may help to enhance the enzymatic digestibility of pretreated wood, but it would lead to wastewater generation.

#### 3.3.3 Acetosolv pulping-alkaline peroxide bleaching pretreatment

The acetosolv pulping process hydrolyzed 19.4 % of the initial cellulose, 88.6 % of the initial hemicellulose, and 91.7 % of the initial lignin was removed during acetosolv pulping and >95.0 % overall delignification was achieved after bleaching of the acetosolv pulp in alkaline hydrogen peroxide solution. The pulping and bleaching process disrupts the lignin and hemicellulosic network and exposes the cellulose, allowing it to be more readily accessed by the enzymes [16]. The enzymatic hydrolysis of BWP by enzyme cocktail released a TRS yield of 74.9 ± 0.8 wt% of THS in 72.0 h and reached ∼95.0 wt% in 144.0 h. The acetosolv pulping-bleaching method produced high-purity pulp (lignin <0.7 wt%) equivalent to a pure cellulosic substrate for enzymatic hydrolysis. However, the presence of 6.2 ± 0.7 wt% hemicellulose in the bleached pulp led to significant resistance for enzymatic hydrolysis by cellulase alone and the TRS yield was only 44.9 wt% of THS in 96.0 h at 46.3 °C, whereas the TRS yield reached 88.9 wt% in the presence of xylanase from the Viscozyme L in the cocktail enzyme (xylose yield increased to 10.0 folds) at 42.5 °C. Also, it is worth noting that the enzyme loading is substantially lower (0.15 FPU cellulase with 2.79 FPU cellulolytic complex enzymes) for bleached wood pulp in comparison to other organosolv pulping methods where a minimum of 15.0 FPU cellulase enzymes have been used to yield similar amount of sugar at similar rates [[61], [62], [63]] (Table 3). The synergy between the two enzymes (cellulase and cellulolytic complex enzymes), as well as the optimization of enzyme amount per gram of substrate and the temperature led to achieving a lower enzyme loading requirement.

**Table 3:** Comparison of organosolv pulping pretreatment method and their enzymatic digestibility by different enzyme loads.

Biomass	Organosolv pretreatment (solvent, catalyst, temperature, time)	Delignification	Enzymatic hydrolysis (substrate concentration, enzyme U/g substrate)	TRS yield by enzymatic hydrolysis	Ref.
*Eucalyptus* wood	Propanol 70.0 % v/v, None, 220.0 °C, 2.0 h	73.9 %	5.0 % solid, 15.0 FPU Cellic@CTec2	79.4 % in 72.0 h	[61]
	Propanol 50.0 % v/v, None, 220.0 °C, 2.0 h	81.3 %		88.6 % in 72.0 h	
Norway spruce	Ethanol 63.0 % v/v, 0.05 M formic acid, 235.0 °C, 1.5 h	65.0 %	1.0 % cellulose, 30.0 FPU Celluclast 1.5 L with 32.0 pNPGU	100.0 % in 48.0 h	[62]
*Eucommia ulmoides* Oliver wood	Ethanol 50.0 % v/v, 1 % HCl, 180.0 °C, 0.5 h	70.4 %	5.0 % solid, 15.0 FPU cellulase	∼88.0 % in 96.0 h	[63]
Spruce	Acetic acid 93.0 % v/v, 0.3 % HCl w/w, 115.0 °C, 3.0 h	>95.0 %	2.0 % solid, 0.15 FPU cellulase with 2.79 FPU cellulolytic complex enzymes	86.9 % in 96.0 h	This study^a^

a Acetic acid pulping was followed by alkaline peroxide bleaching and net delignification was >95.0 %.

It has been found that enzyme works inefficiently in the presence of lignin due to adsorption of enzyme protein on lignin by hydrophobic, electrostatic, and hydrogen bonding interactions which block the enzyme-carbohydrate interaction [55,64,65]. Several lignin-blockers have been shown to be effective in reducing this effect on enzymes, such as surfactants (i.e., TWEEN and poly ethyl glycol) and non-catalytic proteins (i.e., bovine serum albumin, soy protein) [[66], [67], [68]]. For example, Luo et al. (2019) used a soy protein for the hydrothermally pretreated wood biomass that blocked the attachment of enzyme to lignin and enhanced the enzymatic hydrolysis efficiency and TRS yield up to 200 % [67]. Biomass pretreated with scCO_2,_ and alkali-assisted ultrasonic pretreatment methods did not result in lignin and hemicellulose removal which resulted in inefficient enzyme-carbohydrate interaction and no substantial improvement in TRS yield. In contrast, the acetosolv pulping-alkali peroxide method, which is capable of lignin removal, successfully achieved high yield at a very low load (3.0 FPU in total) of enzyme cocktail.

### 3.4 Effect of supercritical CO_2_ pretreatment on enzyme cocktail

**Fig. 5:**
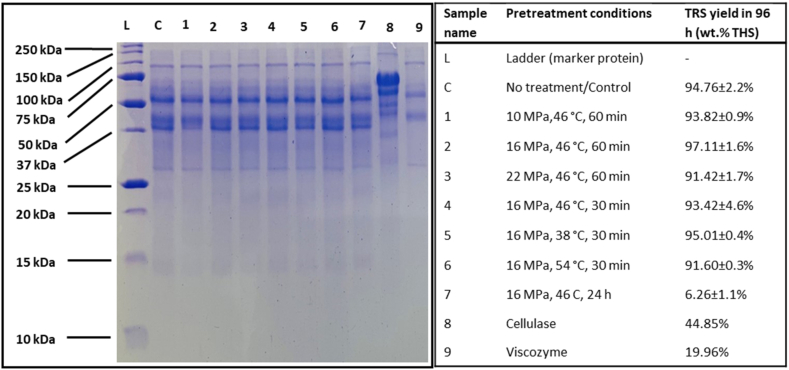
Comparison of untreated and scCO_2_ pretreated enzyme's primary structure analyzed by SDS-PAGE (n = 2) where TRS yield is from enzymatic hydrolysis of BWP by untreated and pretreated enzyme cocktail in 96.0 h.

Under the strategy of exploring the effect of scCO_2_ pretreatment of enzyme, the enzyme cocktail was treated under scCO_2_ and characterized for enzyme activity and structural changes. SDS-PAGE analysis of untreated and scCO_2_ pretreated enzyme cocktail solution showed that there was no breakage or agglomeration in the primary structure of the enzymes (Fig. 5). Namely, the profile of resolved proteins observed for the untreated enzymes were present and maintained the same ratio of intensities in the pretreated enzyme solutions. All enzymes in the cocktail thus appear resilient at the primary structural level to high pressure for up to 24.0 h of static exposure of scCO_2_ at 10.0–22.0 MPa and 38.0–54.0 °C (Fig. 5). The individual enzyme mixtures were also resolved by SDS-PAGE, showing the expected presence of multiple constituents with different molecular weights in both the cellulase and cellulolytic complex enzyme mixtures.

**Fig. 6:**
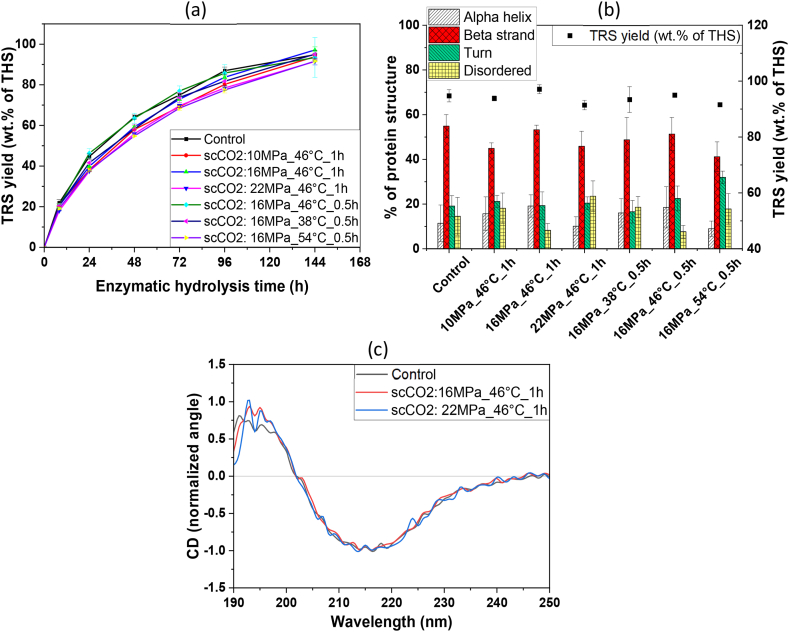
(a) Effect of scCO_2_ pretreatment on TRS yield (n = 2), (b) the enzyme's secondary structure analyzed by FTIR and corresponding TRS yield profile for BWP hydrolysis, and (c) secondary structure analyzed by CD specroscopy (n = 3).

The enzyme cocktail pretreated for short time (0.5–1.0 h) under scCO_2_ at 38.0–54.0 °C and 10.0–22.0 MPa, did not have any significant change in the enzyme activity in comparison to the untreated enzyme for hydrolyzing the bleached wood pulp (BWP). The TRS yield profile in enzymatic hydrolysis with scCO_2_ pretreated enzyme has been shown along with untreated enzyme in Fig. 6a. The protein secondary structure content of the untreated and treated enzyme cocktails was analyzed using both FTIR and CD spectroscopy (Fig. 6b & c). In both cases, the global secondary structural composition is observed, reflecting the overall ensemble-averaged structuring of all the different enzymes (endoglucanase, exoglucanase, and β-glucosidase from cellulase, and xylanases, β-glucanase, pectinases, hemicellulases from Viscozyme L enzyme complex) present in the enzyme cocktail.

Spectral decomposition was carried out for the amide I band of FTIR spectra (Fig. S7), allowing evaluation of protein structural composition for each cocktail. In each case, a mixture of α-helical, β-strand/sheet content, turn, and disordered structuring is observed, with β-strand/sheet being the predominant structural component (Fig. 6b). This is fully consistent with structural expectations for these typically β-sandwich or mixed β-sheet/α-helical enzymes. Based on ANOVA test, the observed differences in secondary structure content are insignificant (p = 0.27). The secondary structure of untreated enzymes as well as the samples with the greatest and least TRS yield were also analyzed using CD spectroscopy. The virtually unchanged far-UV CD spectral lineshape regardless of condition clearly indicates that the treatments did not significantly alter the global secondary structure composition of the enzyme cocktail (Fig. 6c). The single minimum band observed at approximately 216.0 nm is consistent with predominant β-strand/sheet composition in the cocktail [69], following the FTIR-based spectral decomposition.

Although the enzyme cocktail is a mixture of proteins, changes in secondary structuring of individual enzyme components of the mixture upon scCO_2_ treatment will be reflected in spectroscopically observable changes in the global secondary structure composition providing valuable information about cellulolytic enzyme treatment with high pressure and scCO_2_ [[70], [71], [72]]. Herein, FTIR and CD spectroscopy thus independently demonstrate that enzyme's global secondary structuring is not substantially perturbed by pretreatment. Considering the lack of protein truncation observed in SDS-PAGE and the lack of global secondary structural perturbation apparent by both FTIR and CD spectroscopy, this is consistent with the tertiary structure of the enzymes in the cocktail also generally being insensitive to the pretreatment process given consistent hydrolytic activity It is worth mentioning that there might have been changes in the secondary and tertiary structures of individual enzymes, which cannot be observed in a cocktail or mixture of enzymes. Therefore, further separation of enzymes and treatment of individual enzymes will be beneficial to understand the effects on their secondary structures influenced by the scCO_2_ and high pressure [73,74]. Conversely, the enzyme cocktail pretreated with long exposure to scCO_2_ (16.0 MPa, 46.0 °C, 24.0 h) had a substantial reduction in yield from 44.6 ± 1.3 wt% (untreated enzyme) to 6.3 ± 1.1 wt% (pretreated enzyme) of THS at 24.0 h of enzymatic hydrolysis of BWP. Oliveira et al. (2006) and Melgosa et al. (2015) had also observed negative influence on enzyme activity of different lipase enzymes for the long exposure (3.0–6.0 h) at high pressures (25.0–28.0 MPa) of scCO_2_ [75,76]. The reason for this decreased hydrolytic activity could be the structural changes in the microenvironment of enzyme, its active sites, and the low pH due to carbonic acid formation by CO_2_ under high pressure [77]. In contrast to this latter finding, Paljevac et al. (2007) exposed crude cellulase to scCO_2_ (10.0 MPa, 35.0 °C, 24.0 h), and the activity remained at 101.7 % of the untreated enzyme [28]. The influence of exposure time on cross linked enzyme aggregates (CLEAs) of cellulase enzyme was investigated by Hojnik Podrepšek et al. (2019). They found that the short exposure at low pressure of scCO_2_ (10.0 MPa, 40.0 °C, 1.0–3.0 h) treatment had enhanced residual activity (147 %) whereas long exposure of 4.0–24.0 h had residual activity of <80.0 % [30]. The cellulase and lipase enzymes have shown enhanced enzyme activities after scCO_2_ pretreatment. Senyay-Oncel & Yesil-Celiktas investigated the effect of scCO_2_ on cellulase enzyme under 12.0–24.0 MPa, 41.0–67.0 °C for 1.5–2.5 h of pretreatment time. The cellulase activity was increased from 6.25 U/mL (untreated) to 9.27 U/mL (148.3 % residual activity) after scCO_2_ pretreatment at optimum condition (18.0 MPa, 54.0 °C, 2.0 h) and 10.0 g/min CO_2_ flow rate whereas the activity was decreased to a residual activity of 4.61 U/mL at 12.0 MPa, 41.0 °C, 2.5 h and 4.0 g/min flow rate. The study showed that the enzymes have an active conformation at certain optimum condition of scCO_2_ and apart from that the enzyme could be partially inactivated [29]. However, the effect of scCO_2_ on the structure of enzyme for increased or decreased activity was not investigated [29,30]. Since the enzymes are biomolecules and have different primary, secondary, and tertiary level structures, each enzyme may behave differently.

## 4 Conclusions

The focus in LC biorefinery is to explore alternative approaches to traditional chemical pretreatments of lignocellulosic biomass. This study aims to investigate the comparative effectiveness of greener alternatives in enhancing the enzymatic digestibility of spruce wood biomass. Supercritical CO_2_, acetosolv pulping-alkaline peroxide bleaching, and ultrasound-assisted alkaline pretreatment methods were used to disrupt the resilience of spruce wood biomass structure to enhance the enzymatic digestibility for producing reducing sugars. The pretreated solids were hydrolyzed with an enzyme cocktail, a mixture of cellulolytic enzymes at optimized reaction conditions (i.e., enzyme concentration, temperature). The mixture of enzymes (commercial cellulase from *Trichoderma reesei* ATCC 26921 (9.65 mg enzyme/g solid) and cellulolytic enzyme complex Viscozyme L from *Aspergillus* sp. (599.4 mg enzyme/g solid)) showed a TRS yield of ∼95.0 % at 42.5 °C at pH 5.0 in citrate buffer in 144.0 h with degree of synergism of 1.34 between enzymes. Acetosolv pulping followed by alkaline peroxide bleaching was the most effective method among the three pretreatment methods examined in this study. The scCO_2_ (20.0 MPa, 180.0 °C, 1.0 h) pretreatment of wood at high water-solid ratio of 4.0–10.0 mL/g slightly increased the TRS yield from 14.1 ± 0.8 wt% to 19.5 ± 0.8 wt% of THS. However, the improvement in enzymatic digestibility of spruce wood by scCO_2_ was not substantial in comparison to acetosolv pulping method of pretreatment. Furthermore, when subjected to short-term pretreatment under supercritical carbon dioxide (scCO_2_), the enzymes present in the enzyme cocktail exhibited no significant alterations in their primary and secondary structures. This outcome aligns with the observed preservation of enzyme activity during the pretreatment process. However, the activity declined to 25.0 % of untreated enzyme activity for prolonged exposure (24.0 h) under scCO_2_. This study, along with the literature, indicates that enzymes derived from different sources exhibit varying levels of effects (positive, negative, or neutral) on enzyme activity after pretreatment under scCO_2_. The outcomes of this study suggest that the acetosolv pulping-alkali peroxide bleaching method has promising yield from the spruce wood biomass and therefore the study could be extended to its application on various biomass and scaling up to large scale in order to explore industrial potential. Further exploration of cellulolytic enzymes and biomass using greener pretreatment methods, as demonstrated in this study, could aid in achieving the goal of developing sustainable and environmentally friendly methods for utilizing abundantly available lignocellulosic biomass.

## CRediT authorship contribution statement

**Pawan Kumar:** Writing – original draft, Visualization, Methodology, Investigation, Conceptualization. **Azadeh Kermanshahi Pour:** Writing – review & editing, Supervision, Resources, Project administration, Methodology, Funding acquisition. **Satinder Kaur Brar:** Writing – review & editing, Supervision, Resources, Project administration, Funding acquisition. **Chunbao Charles Xu:** Writing – review & editing, Supervision. **Quan Sophia He:** Writing – review & editing. **Sara Evans:** Writing – review & editing, Visualization, Investigation. **Jan K. Rainey:** Writing – review & editing, Visualization, Resources, Data curation.

## Declaration of competing interest

The authors declare that they have no known competing financial interests or personal relationships that could have appeared to influence the work reported in this paper.

## Footnotes

Appendix B Supplementary data to this article can be found online at https://doi.org/10.1016/j.heliyon.2023.e21811.
