# Supplementary material for: Enzymatic digestibility of lignocellulosic wood biomass: Effect of enzyme treatment in supercritical carbon dioxide and biomass pretreatment
Source: Heliyon. 2023 Oct 31;9(11):e21811. doi: 10.1016/j.heliyon.2023.e21811 (PMC10660486; doi:10.1016/j.heliyon.2023.e21811)
Supplement: Multimedia component 1 [file mmc1.docx]

**Supplementary information**

**Enhancement of Enzymatic Digestibility for Fermentable Sugar Production by Pretreatment on Enzyme and Lignocellulosic Biomass**

Pawan Kumar ^a^, Azadeh Kermanshahi Pour ^a*^, Satinder Kaur Brar ^b^, Chunbao Charles Xu ^c^, Quan Sophia He ^d^, Sara Evans ^e^, Jan K. Rainey ^e,f^

*^a^ Biorefining and Remediation Laboratory, Department of Process Engineering and Applied, Science, Dalhousie University, 1360 Barrington Street, Halifax, Nova Scotia B3J 1Z1, Canada*

*^b^ Department of Civil Engineering, Lassonde School of Engineering, York University, North York, Toronto, Ontario M3J 1P3, Canada*

*^c^ School of Energy and Environment, City University of Hong Kong, Hong Kong SAR*

*^d^* *Department of Engineering, Faculty of Agriculture, Dalhousie University, Truro, NS, Canada*

*^e^* *Department of Chemistry, Dalhousie University, Halifax, Nova Scotia B3H 4R2, Canada*

*^f^ Department of Biochemistry & Molecular Biology and School of Biomedical Engineering, Dalhousie University, Halifax, Nova Scotia B3H 4R2, Canada*

1. **Enzyme activity**

The enzyme activity was analyzed using 2 % (w/v) microcrystalline cellulose, MCC (also known as cotton linters) substrate and enzyme concentration 0.050 mg/mL in 2 mL reaction volume. The reaction temperature was 55 °C, pH of 5.0 of 10 mM sodium citrate buffer at shaking speed of 150 rpm.

After enzyme hydrolysis of MCC substrate for 60 min, the enzyme activity was calculated using eq. 1 and eq. 2

Enzyme activity (U/mL) $=\frac{total sugar released (mg)\times\frac{1\mu mol glucose}{0.180 mg glucose}}{Enzyme volume (mL) \times60 min}$ (1)

Specific activity (U/mg enzyme)$=\frac{Enzyme activity \frac{U}{mL}}{Enzyme protein concentration (\frac{mg}{mL})}$ (2)

1. **Enzyme concentration and temperature optimization of individual enzyme**

The enzyme concentration and optimum temperature for both enzymes cellulase and viscozyme were optimized separately using untreated wood substrate. Cellulase and Viscozyme has specific enzyme activities of 0.342 U/mg cellulase at pH 5.0, 55 °C, and 0.046 U/mg Viscozyme at 5.0 and 45 °C. Spruce wood of 0.5-1.0 mm particle size has been used as substrate in both enzymes. The total sugar released in control (without enzyme or without substrate) and in the reaction, was calculated using DNS method. The sugar yield in terms of wt.% of total hydrolyzable sugars (THS) was calculated as below:

Sugar yield (wt.% of THS) = $\frac{total sugar released (mg)}{\frac{cellulose in feedstock (mg)}{0.9}+ \frac{hemicellulose (mg)}{0.88}}\times100$ (3)

Table S**Error! No text of specified style in document.**‑1. Response surface method (RSM) for optimizing individual enzyme (cellulase and cellulolytic enzyme complex viscozyme) concentration and temperature at atmospheric pressure

| **Factors** | **Unit** | **-α** | **-1** | **0** | **+1** | **+α** |
| --- | --- | --- | --- | --- | --- | --- |
| Temperature (X_1_) | (°C) | 36.9 | 40 | 47.5 | 55 | 58.1 |
| Enzyme concentration (X_2_) | (U enzyme/g-wood) | 2.04 | 3.46 | 6.92 | 10.39 | 11.80 |

Table S**Error! No text of specified style in document.**‑2. Design of experiment for optimizing **cellulase** concentration and temperature using for response surface method using 4 factorial, 4 axial, and 4 center points.

| **Run Order** | **Pt Type** | **Temperature (°C)** | **Enzyme**  **(U/g biomass)** | **Sugar yield (wt.% of THS)** |
| --- | --- | --- | --- | --- |
| 1 | -1 | 58.1 | 6.92 | 1.95 |
| 2 | 1 | 40.0 | 3.46 | 2.74 |
| 3 | 0 | 47.5 | 6.92 | 3.59 |
| 4 | 1 | 55.0 | 3.46 | 2.52 |
| 5 | -1 | 47.5 | 2.04 | 2.88 |
| 6 | -1 | 47.5 | 11.80 | 3.49 |
| 7 | 0 | 47.5 | 6.92 | 3.23 |
| 8 | 1 | 55.0 | 10.38 | 2.79 |
| 9 | 0 | 47.5 | 6.92 | 3.49 |
| 10 | 1 | 40.0 | 10.38 | 2.73 |
| 11 | -1 | 36.9 | 6.92 | 2.93 |
| 12 | 0 | 47.5 | 6.92 | 3.59 |

Table S**Error! No text of specified style in document.**‑3. Design of experiment for optimizing individual **cellulolytic enzyme complex viscozyme** concentration and temperature using for response surface method using 4 factorial, 4 axial, and 4 center points.

| **Run Order** | **Pt Type** | **Temperature (°C)** | **Enzyme**  **(U/g biomass)** | **Sugar yield (wt.% of THS)** |
| --- | --- | --- | --- | --- |
| 1 | -1 | 58.1 | 6.92 | 0.42 |
| 2 | 1 | 40.0 | 3.46 | 3.48 |
| 3 | 0 | 47.5 | 6.92 | 4.33 |
| 4 | 1 | 55.0 | 3.46 | 0.51 |
| 5 | -1 | 47.5 | 2.04 | 2.22 |
| 6 | -1 | 47.5 | 11.80 | 9.52 |
| 7 | 0 | 47.5 | 6.92 | 5.71 |
| 8 | 1 | 55.0 | 10.38 | 0.55 |
| 9 | 0 | 47.5 | 6.92 | 3.76 |
| 10 | 1 | 40.0 | 10.38 | 5.95 |
| 11 | -1 | 36.9 | 6.92 | 5.33 |
| 12 | 0 | 47.5 | 6.92 | 3.62 |

The enzymatic hydrolysis reaction at atmospheric condition were conducted for 72 h and samples were taken at 0, 2, 4, 8, 16, 24, 48, 72 h. A 300 µL from the reactions was taken at sampling times and diluted three time by adding 600 µL distilled water. The enzymatic reaction was stopped by heating the diluted solution in water bath (100 °C) for 5 min. The hydrolysates were analyzed by HPLC for the sugar released in the hydrolysate.

The RSM analysis showed that the purified cellulase from *Trichoderma reesei* ATCC 26921, has optimum temperature between 45-48 °C at 7-9 activity U of enzyme per g of wood substrate in 10 mM citrate buffer. In the chosen range of temperature and enzyme concentration, Temperature has the major significant effect. The optimized condition of cellulase was 8.31 U/g wood at 46 °C (*Figure 1*). The RSM analysis of viscozyme (from *Aspergillus sp.*) showed that the viscozyme enzyme has optimum temperature ~37-42 °C at enzyme concentration of above 11 activity U per g of wood substrate in 10 mM citrate buffer solution. The optimum condition of viscozyme was above 11.61 U/g wood at 37 5 °C (*Figure 2*).


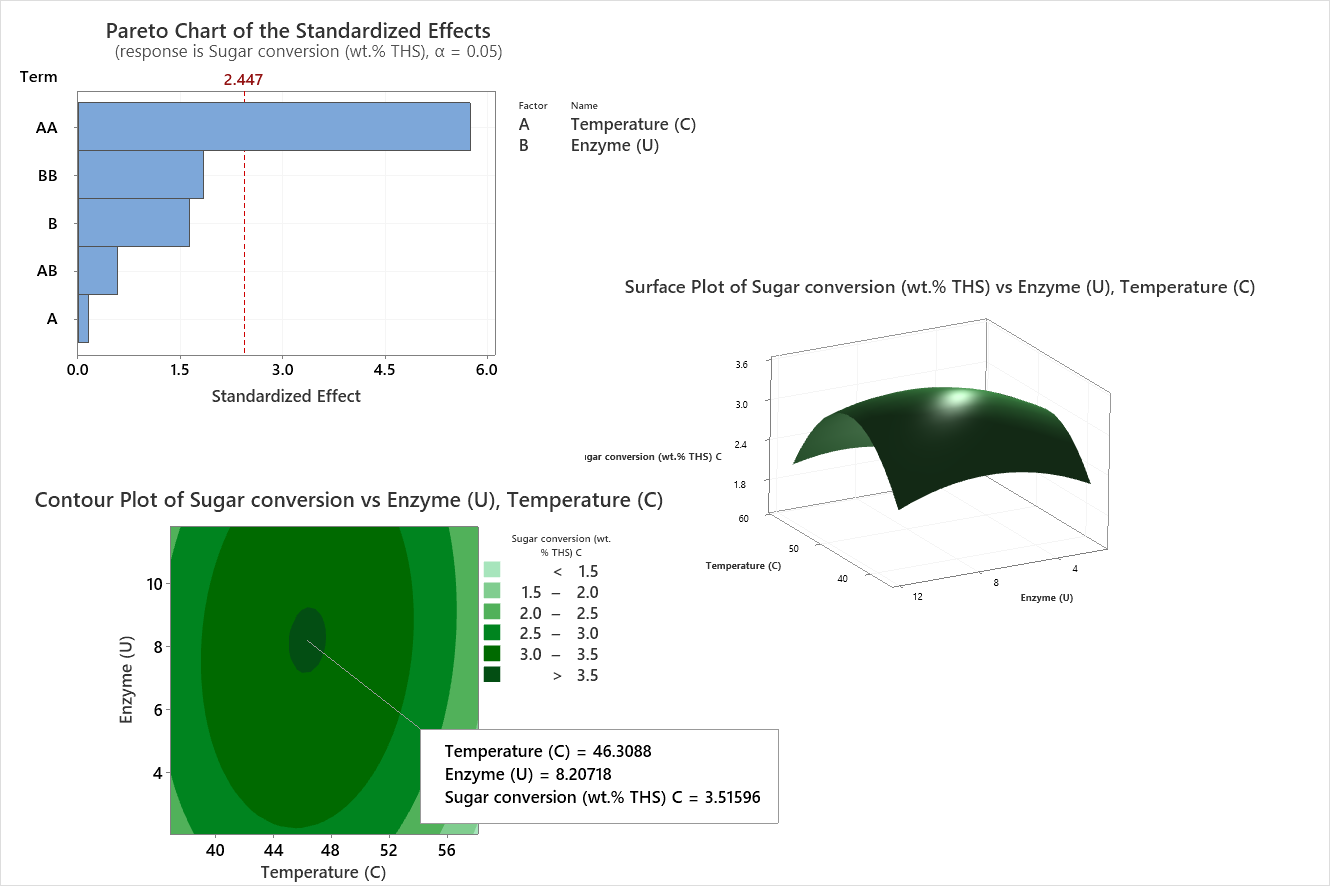


Figure S1. Pareto, surface, and contour plot of the response surface method of optimization model for purified cellulase enzyme (n=4 center points)

Table S**Error! No text of specified style in document.**‑4. Analysis of Variance for the cellulase enzyme.

| **Source** | **DF** | **Adj SS** | **Adj MS** | **F-Value** | **P-Value** |
| --- | --- | --- | --- | --- | --- |
| Model | 5 | 2.41852 | 0.48370 | 8.36 | **0.011** |
| Linear | 2 | 0.15489 | 0.07744 | 1.34 | 0.331 |
| Temperature (C) | 1 | 0.00138 | 0.00138 | 0.02 | 0.882 |
| Enzyme (U) | 1 | 0.15351 | 0.15351 | 2.65 | 0.154 |
| Square | 2 | 1.94303 | 0.97152 | 16.79 | 0.003 |
| Temperature (C)*Temperature (C) | 1 | 1.91400 | 1.91400 | 33.08 | 0.001 |
| Enzyme (U)*Enzyme (U) | 1 | 0.19475 | 0.19475 | 3.37 | 0.116 |
| 2-Way Interaction | 1 | 0.01960 | 0.01960 | 0.34 | 0.582 |
| Temperature (C)*Enzyme (U) | 1 | 0.01960 | 0.01960 | 0.34 | 0.582 |
| Error | 6 | 0.34717 | 0.05786 |  |  |
| Lack-of-Fit | 3 | 0.26047 | 0.08682 | 3.00 | 0.195 |
| Pure Error | 3 | 0.08670 | 0.02890 |  |  |
| Total | 11 | 2.76569 |  |  |  |


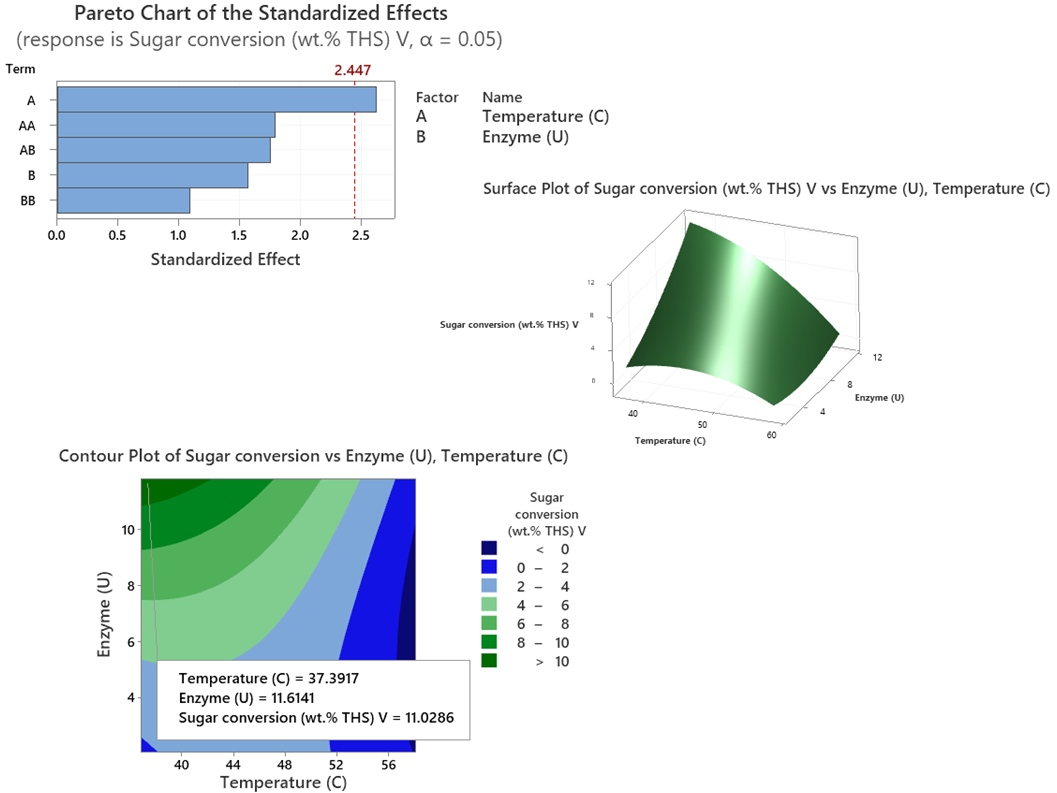


Figure S2. Pareto, surface, and contour plot of the response surface method of optimization model for cellulolytic enzyme mixture Viscozyme (n=4 center points)

Table S**Error! No text of specified style in document.**‑5. Analysis of Variance of viscozyme enzyme.

| Analysis of Variance |  |  |  |  |  |
| --- | --- | --- | --- | --- | --- |
| Source | DF | Adj SS | Adj MS | F-Value | P-Value |
| Model | 5 | 79.9723 | 15.9945 | 9.9 | 0.007 |
| Linear | 2 | 15.0793 | 7.5396 | 4.67 | 0.06 |
| Temperature (C) | 1 | 11.1043 | 11.1043 | 6.87 | 0.04 |
| Enzyme (U) | 1 | 3.9749 | 3.9749 | 2.46 | 0.168 |
| Square | 2 | 8.6814 | 4.3407 | 2.69 | 0.147 |
| Temperature (C)*Temperature (C) | 1 | 5.1589 | 5.1589 | 3.19 | 0.124 |
| Enzyme (U)*Enzyme (U) | 1 | 1.9278 | 1.9278 | 1.19 | 0.317 |
| 2-Way Interaction | 1 | 4.9506 | 4.9506 | 3.06 | 0.131 |
| Temperature (C)*Enzyme (U) | 1 | 4.9506 | 4.9506 | 3.06 | 0.131 |
| Error | 6 | 9.6963 | 1.616 |  |  |
| Lack-of-Fit | 3 | 8.8501 | 2.95 | 10.46 | 0.043 |

1. **Optimization enzyme concentrations and temperature of cocktail enzyme**

Further, the enzyme concentration (cellulase 5-15 U/g wood and viscozyme 10-30 U/g wood) and temperature (30-50 °C) were optimized for the mixture of cellulase and viscozyme enzymes based on the optimized condition of cellulase (8.31 U/g wood at 46 °C) and viscozyme (11.61 U/g wood at 37 5 °C). The cellulase and viscozyme enzymes were mixed to make enzyme cocktail. The concentrations of the two enzymes and the temperature were optimized by RSM where concentration of enzymes and temperature were the factors and total sugar yield (wt.% of THS) was the response as shown in Table 4.

Table S**Error! No text of specified style in document.**‑6. Response surface optimization model for enzyme concentration and temperature of combination of cellulase and viscozyme enzymes (n=4)

| **Cellulase (U/g-wood)** | **Viscozyme (U/g-wood)** | **Temperature (****°C)** | **Sugars (C5+C6) wt.% of THS** | **Xylose (C6) wt.% of THS** | **Glucose (C5) wt.% of THS** |
| --- | --- | --- | --- | --- | --- |
| 7.50 | 17.00 | 29.89 | 4.60 | 2.92 | 1.68 |
| 5.00 | 10.00 | 35.00 | 8.78 | 6.10 | 2.68 |
| 10.00 | 10.00 | 35.00 | 7.50 | 5.08 | 2.42 |
| 5.00 | 24.00 | 35.00 | 14.97 | 9.64 | 5.33 |
| 10.00 | 24.00 | 35.00 | 11.53 | 7.40 | 4.13 |
| 7.50 | 17.00 | 42.50 | 15.10 | 9.62 | 5.48 |
| 11.70 | 17.00 | 42.50 | 13.07 | 9.03 | 4.05 |
| 7.50 | 28.77 | 42.50 | 14.46 | 9.85 | 4.62 |
| 7.50 | 17.00 | 42.50 | 13.00 | 8.97 | 4.03 |
| 7.50 | 17.00 | 42.50 | 12.30 | 8.44 | 3.83 |
| 7.50 | 5.23 | 42.50 | 6.79 | 4.92 | 1.86 |
| 7.50 | 17.00 | 42.50 | 12.85 | 7.99 | 4.86 |
| 3.30 | 17.00 | 42.50 | 12.17 | 7.83 | 4.34 |
| 10.00 | 10.00 | 50.00 | 9.56 | 6.26 | 3.30 |
| 10.00 | 24.00 | 50.00 | 16.33 | 10.56 | 5.76 |
| 5.00 | 24.00 | 50.00 | 14.39 | 9.02 | 5.36 |
| 5.00 | 10.00 | 50.00 | 8.35 | 5.48 | 2.87 |
| 7.50 | 17.00 | 55.11 | 4.52 | 2.77 | 1.75 |

Based on the above observation of optimum temperature and enzyme concentration, cellulase concentration (Factor A) in rage of 5-10 U/g wood, and Viscozyme concentration (Factor B) in range of 10-24 U/g, and the temperature (Factor C) in range of 35-50 °C wood will be used for used to determine the optimum condition of enzyme mixture concentration and Temperature for the sugar conversion of biomass.

The enzymatic reaction of mixed enzyme system is completed and analyzed with the Minitab software for analysis of variance (ANOVA) and response optimum conditions to validate the model. Below are the ANOVA and validation conditions in *Table 7*.

Table S**Error! No text of specified style in document.**‑7. Analysis of Variance for sugar conversion in 72 h of enzymatic digestion of untreated spruce wood powder

| **Source** | **DF** | **Adj SS** | **Adj MS** | **F-Value** | **P-Value** |
| --- | --- | --- | --- | --- | --- |
| Model | 9 | 198.361 | 22.0401 | 6.34 | **0.008** |
| Linear | 3 | 96.917 | 32.3058 | 9.30 | 0.006 |
| Cellulase (U/g-wood) | 1 | 0.000 | 0.0002 | 0.00 | 0.994 |
| **Viscozyme (U/g-wood)** | **1** | **94.525** | **94.5253** | **27.21** | **0.001** |
| Temperature (C) | 1 | 2.392 | 2.3919 | 0.69 | 0.431 |
| Square | 3 | 92.608 | 30.8692 | 8.89 | 0.006 |
| Cellulase (U/g-wood)*Cellulase (U/g-wood) | 1 | 0.960 | 0.9602 | 0.28 | 0.613 |
| Viscozyme (U/g-wood)*Viscozyme (U/g-wood) | 1 | 2.337 | 2.3369 | 0.67 | 0.436 |
| Temperature (C)*Temperature (C) | 1 | 83.815 | 83.8150 | 24.13 | 0.001 |
| 2-Way Interaction | 3 | 8.836 | 2.9454 | 0.85 | 0.506 |
| Cellulase (U/g-wood)*Viscozyme (U/g-wood) | 1 | 0.256 | 0.2556 | 0.07 | 0.793 |
| Cellulase (U/g-wood)*Temperature (C) | 1 | 7.742 | 7.7421 | 2.23 | 0.174 |
| Viscozyme (U/g-wood)*Temperature (C) | 1 | 0.839 | 0.8385 | 0.24 | 0.636 |
| Error | 8 | 27.792 | 3.4740 |  |  |
| Lack-of-Fit | 5 | 23.260 | 4.6520 | 3.08 | 0.192 |
| Pure Error | 3 | 4.532 | 1.5106 |  |  |
| Total | 17 | 226.153 |  |  |  |

The ANOVA analysis of the RSM model showed that the model is significant with viscozyme as the influencing factor for maximizing the sugar conversion. It could be observed in the contour and surface response plot of viscozyme and cellulase enzyme that the increasing concentration of cellulase has no significant effect whereas the increase in the viscozyme concentration lead to increase in the sugar conversion (*Figure 3* and *Figure 4*). Effect of temperature with respect to two enzymes, 36 °C to 48 °C is the most effective range of the temperature for the mixed enzyme system with optimum at 42.5 °C.


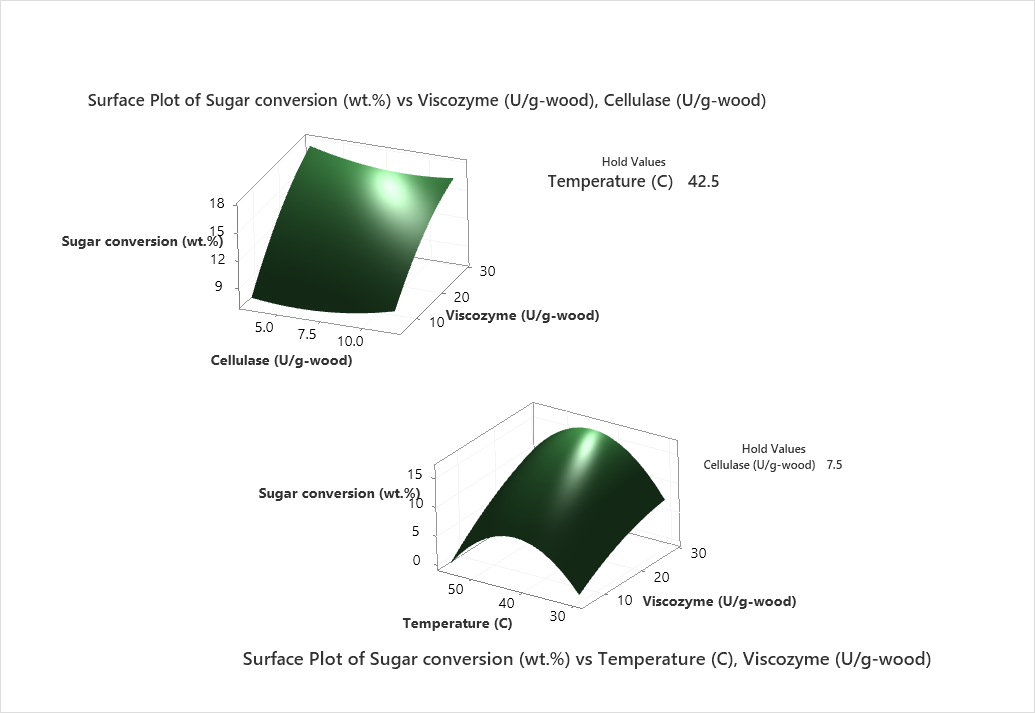


Figure S3. Surface response plot of sugar conversion with respect to viscozyme, cellulase, and temperature in enzyme cocktail at atmospheric pressure reaction.

**
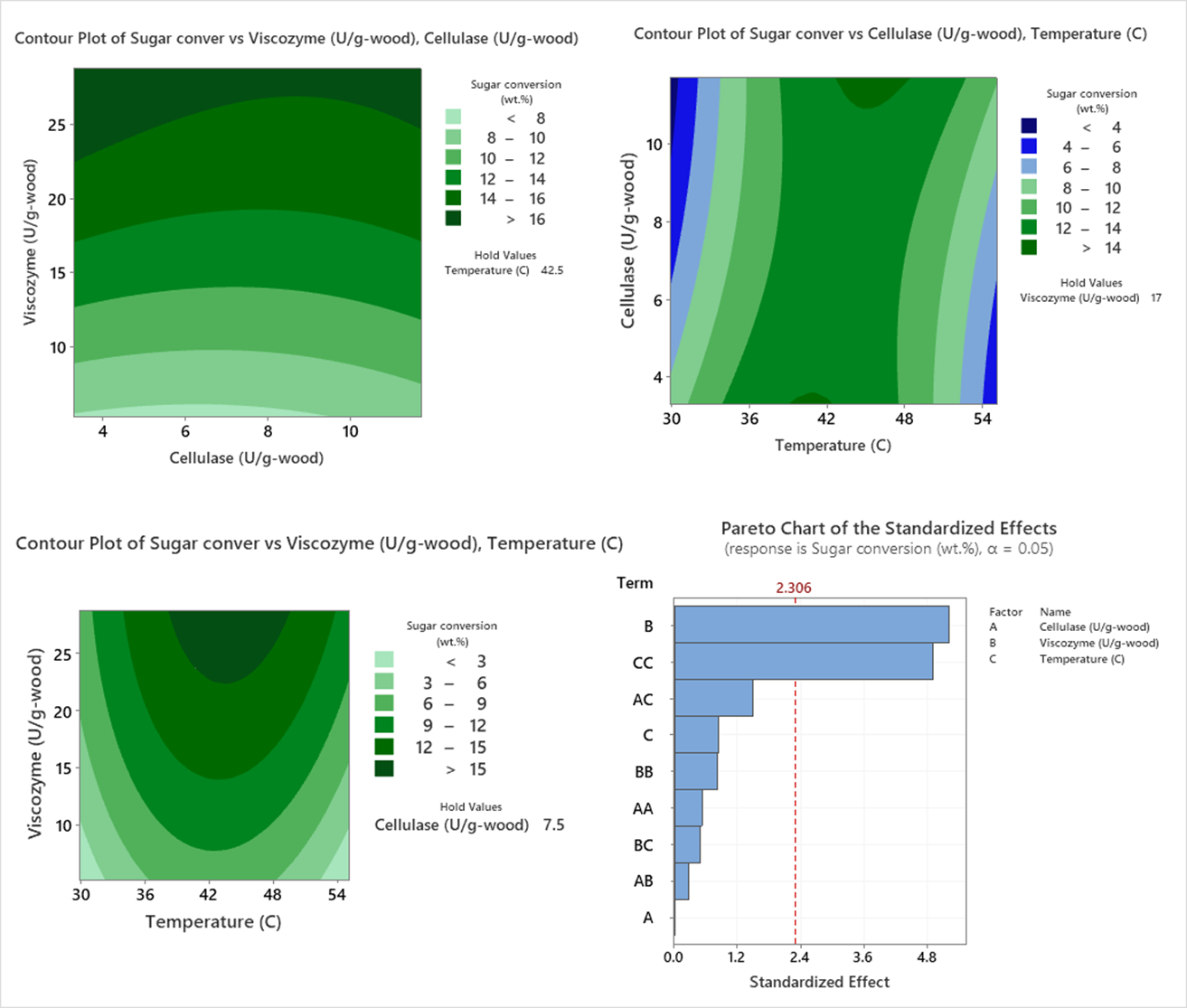
**

Figure S4. Surface response of cellulase, viscozyme and temperature variable for sugar conversion

The optimized reaction condition of the mixed enzyme system for untreated spruce wood hydrolysis is listed in the *Table 8* below:

Table S**Error! No text of specified style in document.**‑8. Optimized concentration of enzymes in cocktail enzyme for 200 mg substrate in 10 mL citrate buffer

| **Components (stock concentration)** | **Amount (in 10 mL citrate buffer (10 mM, pH 5.0))** |
| --- | --- |
| Spruce wood biomass | 200.00 mg |
| Cellulase (14.69 mg/mL) | 9.7 mg enzyme/g wood (or 0.132 mL) |
| Viscozyme (220 mg/mL) | 598.4 mg enzyme/g wood (or 0.544 mL) |
| Sodium Azide (0.002% w/v) | 0.04 mL |
| Buffer (50 mM) | 2 mL |
| Water | 7.284 mL |

**Effect of supercritical CO_2_ pretreatment of cornstalk**

Figure S5. Effect of scCO_2_ on enzymatic digestibility of cornstalk agricultural biomass

**Influence of initial sugar concentration in enzyme cocktail**

The presence of sugars in the commercial Viscozyme L enzyme introduces a significant 141.9 mg/L sugar concentration in the enzyme cocktail. However, the effect of sugar removal was investigated to analyze its influence on the enzymatic activity. Therefore, enzyme cocktail was purified using a spin concentrator and used to hydrolyze the cellulosic substrate (bleached wood pulp). Total protein content of enzyme cocktail decreased to 51.75 from 102.92 mg per 10 mL cocktail solution along with 90-95% initial sugar concentration. There was no significant loss in enzyme activity of the enzyme cocktail (Table S9 Fig. S6).

Figure S6. Influence of initial sugar concentration on enzyme activity

Table S9. Enzyme cocktail purification using spin concentrator to remove sugars

| Sample | Protein mass (mg/mL) | Sugar mass (mg/mL) |
| --- | --- | --- |
| Enzyme cocktail original | 10.3 | 14.1 |
| Enzyme cocktail purified | 5.2 | 1.0 |
| Filtration waste | 5.1 | 13.8 |

**Secondary structure analyses**

**Fourier transform infrared (FTIR)** spectra were collected using a liquid nitrogen-cooled Nicolet iZ10 spectrometer (Thermo Fisher Scientific) equipped with a ConcentratorIR2 Multiple Refraction Attenuated Total Reflection (ATR) attachment with a Silicon ATR crystal (Harrick Scientific Products Inc.) (32 scans, 4.000 cm-1 resolution, range of 4000 to 700 cm-1) at room temperature (22.5 +/- 2.5 ˚C). Data collection and analysis were performed using the software Omnic version 9.11.745 (Thermo Fisher Scientific). Following baseline correction, the amide I region (1600 – 1700 cm-1) was deconvoluted to evaluate protein secondary structure.







Figure S7. FTIR deconvoluted spectra of untreated and scCO_2_ pretreated enzyme cocktail enzyme

**Circular dichroism (CD)** spectra were collected on an Olis DSM20 Circular Spectrophotometer (Bogart, GA) with integration time determined as a function of High Volts in Olis SpectralWorks Version 5.888.272. CD spectra were acquired from 270-180 nm with a 1 nm step size using quartz cuvettes of 0.01 mm path length (Hellma Canada Limited; Concord, ON). Spectra were obtained by averaging three individual scans. The spectra of 20 mM sodium citrate blanks were measured before the samples and were subtracted from the 0.1 mg/mL protein sample CD spectra. The spectra were normalized to their respective minima.
